# Facile synthesis of uniform large-sized InP nanocrystal quantum dots using tris(*tert*-butyldimethylsilyl)phosphine

**DOI:** 10.1186/1556-276X-7-93

**Published:** 2012-01-30

**Authors:** SoMyoung Joung, Sungwoo Yoon, Chang-Soo Han, Youngjo Kim, Sohee Jeong

**Affiliations:** 1 Nanomechanical Systems Research Division, Korea Institute of Machinery and Materials, Daejeon 305-343, Republic of Korea; 2 Department of Chemistry, Chungbuk National University, 52 Naesudong-ro, Heungdeok-gu, Cheongju, Chungbuk 361-763, Republic of Korea; 3 School of Mechanical Engineering, Korea University, Anam-dong, Seongbuk-gu, Seoul 136-713, Republic of Korea

**Keywords:** phosphorus precursor, indium phosphide nanocrystal quantum dot, colloidal synthesis, nontoxic

## Abstract

Colloidal III-V semiconductor nanocrystal quantum dots [NQDs] have attracted interest because they have reduced toxicity compared with II-VI compounds. However, the study and application of III-V semiconductor nanocrystals are limited by difficulties in their synthesis. In particular, it is difficult to control nucleation because the molecular bonds in III-V semiconductors are highly covalent. A synthetic approach of InP NQDs was presented using newly synthesized organometallic phosphorus [P] precursors with different functional moieties while preserving the P-Si bond. Introducing bulky side chains in our study improved the stability while facilitating InP formation with strong confinement at a readily low temperature regime (210°C to 300°C). Further shell coating with ZnS resulted in highly luminescent core-shell materials. The design and synthesis of P precursors for high-quality InP NQDs were conducted for the first time, and we were able to control the nucleation by varying the reactivity of P precursors, therefore achieving uniform large-sized InP NQDs. This opens the way for the large-scale production of high-quality Cd-free nanocrystal quantum dots.

## Introduction

Colloidal III-V semiconductor nanocrystals have attracted interest within the decade due to their less ionic lattice and reduced toxicity compared to II-VI compounds [1-4]. However, the study and application of III-V semiconductor nanocrystals are limited by the difficulty in their synthesis. It is very difficult to obtain a controllable nucleation because the molecular bonds in III-V semiconductors are more covalent [1-4]. The synthesis of InP nanocrystals is the most extensively studied, but until now, InP nanocrystals synthesized by current chemical methods did not achieve the same quality as that of most II-VI semiconductor nanocrystals.

Typical synthetic approaches for III-V nanocrystal quantum dots [NQDs] in a coordinating solvent are adaptations of the method for the II-VI group. However, the common coordinating solvents and ligands and the similar precursors for the II-VI system do not work well for the synthesis of III-V NQDs. Both nucleation and crystal growth processes in these approaches needed long reaction times, all together 2 to 7 days, to yield a crystalline particle [1-4]. Some researchers reported an approach using fatty acids and a mixture of fatty acids and amines and applying a non-coordinating solvent, 1-octadecene [ODE] [5-8]. This method provided a fast, controllable reaction and generated much higher-quality InP nanocrystals. In all cases, the use of the tris(trimethylsilyl)phosphine [P(SiMe_3_)_3_] is mandatory, limiting the control of the synthesis of InP nanocrystals. Other than P(SiMe_3_)_3_, *in situ*-generated PH_3 _was introduced as a P precursor for InP synthesis by Peter Reiss's group [9]. In the synthesis of high-quality InP or InP/ZnS NQDs in terms of size uniformity and crystallinity, a choice of an indium [In] precursor is flexible, but that of a phosphorus [P] precursor is quite limited, mostly relying on a highly expensive, toxic, flammable P source such as P(SiMe_3_)_3_.

In this study, we designed and synthesized various organometallic P precursors with different functional moieties while preserving the P-Si bond. Introducing bulky side chains in our study improved the stability while facilitating InP formation with strong confinement at a readily low temperature regime (210°C to approximately 300°C) by controlling nucleation in the reaction. We therefore were able to obtain a facile synthetic route, achieving highly uniform large-sized InP NQDs. Further growing a shell of a large bandgap material, ZnS, around each core particle using a single-source precursor resulted in highly luminescent NQDs in the entire visible range (560 nm to 640 nm) where their quantum yield [QY] range from 18% to 28%.

## Experimental details

All reagents were purchased from Sigma-Aldrich Corporation (St. Louis, MO, USA) and used without further purification. Reactions were performed in an inert atmosphere.

### Materials

All manipulations were carried out under a dinitrogen atmosphere using standard Schlenk and glovebox techniques. Indium(III) acetate [In(OAc)_3_] (99.99%), myristic acid [MA] (95%), oleic acid [OLA], ODE (90%), and zinc diethyldithiocarbamate [ZDC] were purchased from Sigma-Aldrich and used without further purification. Toluene, hexane, diethylether, and tetrahydrofuran [THF] were dried with sodium/benzophenone ketyl, and methylene chloride was distilled from CaH_2_. All solvents were stored over activated 3-Å molecular sieves [10-12]. All deuterium solvents were dried over activated molecular sieves (3 Å) and were used after vacuum transfer to a Schlenk tube equipped with a J. Young valve [10-12]. P(SiMe_3_)_3 _(**1**) was synthesized using the procedure in the literature [13]. The slightly modified literature method was analogously employed in preparing P(SiMe_2_-*tert*-Bu)_3 _(**2**).

### Synthesis of tris(*tert*-butyldimethylsilyl)phosphine (P(SiMe_2_-*tert*-Bu)_3_) (2)

A solid mixture of sodium (0.32 g, 14 mmol) and potassium (0.41 g, 10.5 mmol) in a two-necked Schlenk flask connected by a reflux condenser was suspended and stirred at 120°C without any solvents for several hours. Sodium and potassium were completely melted, and a mercury-like gray alloy was formed. At an ambient temperature, 50 ml of dimethoxyethane and red phosphorus (0.22 g, 7.0 mmol) were added to a flask containing the sodium/potassium alloy. The reaction mixture was slowly allowed to warm to 50°C and stirred vigorously for 24 h. To the resulting solution, *tert*-butyldimethylchlorosilane (3.48 g, 23.1 mmol) in 20 ml of dimethoxyethane was added via cannula and heated at reflux for another 24 h. The reaction mixture was allowed to cool to room temperature, and then, the volatiles were evaporated under vacuum, leaving a yellow oil to which 15 ml of THF was added. The colorless solution was filtered, and the solvents evaporated to dryness to afford **2 **as a white solid (0.8 g, 30.8%).

The boiling point of **2 **was observed at 108°C to 110°C under the pressure of 10^-3 ^Torr. A solution ^1^H nuclear magnetic resonance [NMR] spectrum (400.13 MHz) at C_6_D_6 _solvent gave only two peaks at 0.34 and 1.04 ppm, which were assigned to Si*Me*_2 _and C*Me*_3_, respectively. Because of the coupling with phosphorus and protons, all peaks were split as doublets with the coupling constants of 3.6 Hz and 0.4 Hz, respectively. ^13^C{^1^H} NMR spectrum (100.61 MHz) at the same deuterium solvent gave three peaks at 1.7, 20.3, and 27.8 ppm, which were associated with Si*Me*_2_, *C*Me_3_, and C*Me*_3_, respectively. Like ^1^H NMR spectrum, all peaks are split as doublets with the coupling constants of 5.03, 17.02, and 3.00 ppm. ^31^P{^1^H} NMR (161.98 MHz) spectrum gave only one peak at 30.05 ppm. Electron impact mass spectrometry [EI-MS] showed the molecular peak of compound **2 **at m/z = 376, and the elemental analysis of **2 **contained 57.25 wt.% C and 12.16 wt.% H, corresponding to a molecular formula of C_18_H_45_PSi_3_.

### Synthesis of tris(dimethylphenylsilyl)phosphine (P(SiMe_2_Ph)_3_) (3)

The desired product **3 **was prepared from sodium (0.32 g, 14 mmol), potassium (0.41 g, 10.5 mmol), red phosphorus (0.217 g, 7 mmol), and dimethylphenylchlorosilane (3.94 g, 23 mmol) in a yield of 67.1% (2.05 g) in a manner analogous to the procedure for **2**.

In the ^1^H NMR (400.13 MHz) spectrum of **3 **at C_6_D_6 _solvent, peaks at 0.30 and 7.57 to 7.15 ppm were observed. Like compound **2**, the peak at 0.30 ppm originated from Si*Me*_2 _was split as doublets with the coupling constant of 6.0 Hz. As expected, one aliphatic carbon at 1.93 ppm and four aromatic carbons peaks at 128.31, 130.50, 133.37, and 136.38 ppm were observed in the ^13^C{^1^H} NMR (100.61 MHz) spectrum. ^31^P{^1^H} NMR (161.98 MHz) spectrum gave only one peak at 24.38 ppm. EI-MS showed the molecular peak of compound **3 **at m/z = 437, and the elemental analysis of **3 **contained 65.82 wt.% C and 7.55 wt.% H, corresponding to a molecular formula of C_24_H_33_PSi_3_.

### Synthesis of indium phosphide NQDs

**Figure 1:**
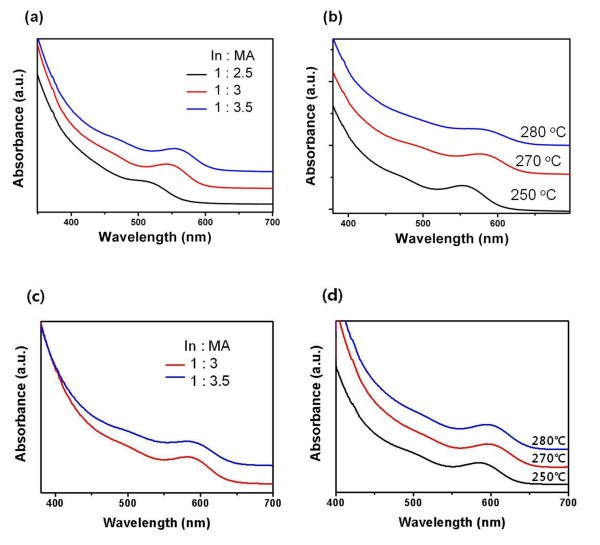
**Absorption spectra of InP NQDs**. (**a**) InP NQDs synthesized by varying the ratio of In and MA by 1:2.5, 1:3, and 1:3.5 using P(SiMe_3_)_3_; (**b**) InP NQDs synthesized at different reaction temperatures (In/MA = 1:3); (**c**) InP NQDs synthesized by varying the ratio of indium and MA by 1:3 and 1:3.5 using P(SiMe_2_-*tert*-Bu)_3_; and (**d**) InP NQD synthesized at different reaction temperatures (In/MA = 1:3).

For a typical experiment of InP NQDs('standard reaction'; Figure 1), 0.04 mmol of In(OAc)_3 _was added to a mixture of 0.12 mmol MA and 4 ml ODE in a 50-ml three-necked flask. The solution was then heated to 110°C for 1.5 h in vacuum. Injection solution was prepared by dissolving 0.02 mmol of P(SiMe_3_)_3_, P(SiMe_2_-*tert*-Bu)_3_, or P(SiMe_2_Ph)_3 _in 1 ml of ODE while the mixture was still degassed from the reaction vessel. Then, the solution was injected rapidly into the hot reaction solution at 270°C under N_2. _After injection, the solution was cooled. The products were precipitated and washed with methanol and 1-butanol and re-dispersed in hexane.

### Synthesis of InP/ZnS

For *in situ *fabrication of InP/ZnS core-shell NQDs, the temperature of the growth solution of InP was lowered after a 10-min growth to 200°C. Thirty milligrams of ZDC, 1 ml of TOP, and 1 ml of OLA were added to 1 ml of ODE in nitrogen atmosphere. After injection, the solution was cooled to room temperature and the solutions were precipitated with an excess of methanol and 1-butanol and dried in air.

### Optical and structural characterizations

The microstructure and crystallographic structures were investigated by field emission transmission electron microscopy (Tecnai F30 Super-Twin, FEI Co., Hillsboro, OR, USA; Yun-Chang Park, KAIST NanoFab). The absorption and photoluminescence were characterized with a UV-visible [Vis] spectrophotometer (SD-1000, Scinco Co., Ltd., Gangnam-gu, Seoul, South Korea) and a fluorometer (Fluorolog, Horiba Jobin Yvon Inc., Edison, NJ, USA).

^1^H, ^13^C{^1^H}, and ^31^P{^1^H} NMR spectra were recorded at ambient temperature on a Bruker DPX-400 NMR spectrometer (Bruker Optik GmbH, Ettlingen, Germany) using standard parameters. The chemical shifts are referenced to the residual peaks of C_6_D_6 _(*δ *7.15, ^1^H NMR; *δ *128.0, ^13^C{^1^H} NMR). EI-mass spectra were obtained from Korea Basic Science Institute.

## Results and discussion

**Figure 2:**
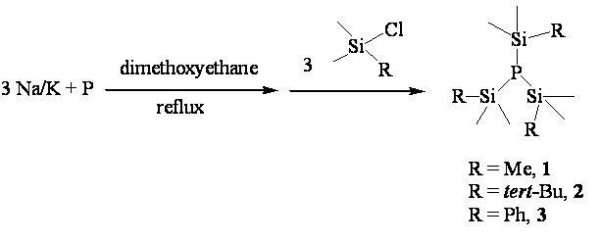
**Scheme 1: Synthesis of various phosphorus precursors 1 to 3**.

We designed the P precursors for InP synthesis while preserving the P-Si bond. The bond dissociation energy of the P-Si bond is 363.6 KJ/mol which is easier to cleave compared to the P-C bond (507.5 KJ/mol). We introduced various side chains to enhance stability, thereby easing the control of the nucleation process during the reaction. Scheme 1 (Figure 2) shows the facile synthesis of designed phosphorus precursors such as P(SiMe_3_)_3 _(**1**), P(SiMe_2_-*tert*-Bu)_3 _(**2**), and P(SiMe_2_Ph)_3 _(**3**). Precursors **1 **to **3 **were synthesized using the modified procedure in the literature [13]. Specifically, the treatment of a mixture of Na/K alloy and red phosphorus with RSiMe_2_Cl (R = Me, *tert*-Bu, and Ph) in dimethoxyethane gave, after workup, a novel P(SiMe_2_R)_3 _(R = Me, **1**; R = *tert*-Bu, **2**; R = Ph, **3**) as colorless compounds in 30% to 70% isolated yield as shown in Scheme 1. Compounds **1 **and **3 **were isolated as colorless oil; however, compound **2 **was obtained as a colorless solid. Compound **1 **was pyrophoric and air-sensitive, but compounds **2 **and **3 **were stable in air for a few hours, and they were slightly decomposed in the C_6_D_6 _solutions in capped NMR tubes at room temperature after a few days and were easy to handle. They are soluble in aromatic solvents and in polar organic solvents. All compounds have been fully characterized by various spectroscopic data and EI-mass analysis. The ^1^H NMR spectra of **1 **to **3 **display well-defined resonances with their expected integrations. Upon complexation to phosphorus, the proton resonances of the methyl, *tert*-butyl, or phenyl attached to silicon are shifted downfield relative to those of corresponding silicon precursors. Also, all protons in complexes **1 **to **3 **were split as doublet due to the presence of coupling between P and H. In the case of **1**, the greater extent of downfield shifts for the methyl protons than for the *tert*-butyl protons upon complexation, suggesting a strong interaction between the P and H in methyl groups and a weak interaction between the P and H in *tert*-butyl groups. The purities of **1 **to **3 **were checked by ^31^P NMR spectroscopy, which revealed only one peak near 30 ppm.

**Figure 3:**
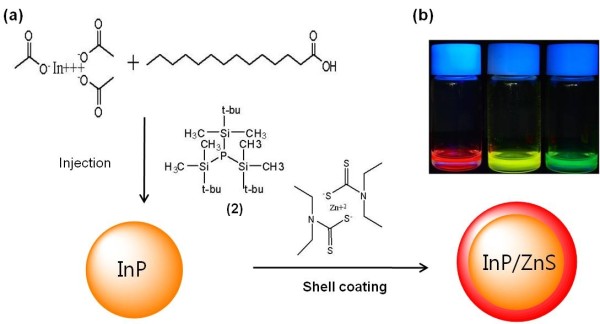
**One-pot synthesis of core-shells and their optical properties**. (**a**) One-pot synthesis of InP with P(SiMe_2_-*tert*-Bu)_3 _followed by ZnS shell overcoating. (**b**) Size-tunable emission from InP/ZnS NQDs synthesized using a new P precursor (red-green).

We started our experiments with a synthesis of the InP NQDs using compounds **2 **(Figure 3a) and **3**. For a typical experiment, 0.04 mmol of In(OAc)_3 _was added to a mixture of 0.12 mmol of MA and 4 ml of ODE in a 50-ml three-necked flask. The solution was then heated to 110°C for 1.5 h in vacuum. Injection solution was prepared by 0.02 mmol of compounds **2 **or **3 **in 1 ml of ODE while the mixture was still degassed from the reaction vessel. Then, the solution was injected rapidly into the hot reaction solution at 250°C under N_2_. When we prepared InP NQDs using compound **3**, we obtained a colorless solution and the resulting absorption spectrum did not show any distinct peak from an excitonic transition. On the other hand, compound **2 **which contains the *tert*-butyl group underwent the NQD formation. We further developed a synthetic route of InP NQDs using P(SiMe_2_-*tert*-Bu)_3. _The effects of different experimental parameters on the NQDs' growth were studied, including the reaction temperature, the ratios, and the concentrations of precursors. First, we studied the synthetic scheme forming InP nanocrystals with the P(SiMe_3_)_3 _precursor. The ratio range of indium to acid was varied from 1:2.5 to 1:3.5 to optimize the reaction condition. As suggested by other groups, MA is known to act as a ligand which allowed a balanced nucleation and growth rate desired for the growth of relatively monodisperse InP NQDs, resulting in uniform NQDs in the visible range [14]. Figure 1a shows that when the molar ratio of In/MA in the solution was 1:3, the reaction generated InP nanocrystals with a good size distribution indicated by the well-distinguished absorption features. When this molar ratio was varied to 1:2.5, the reaction generated nanocrystals without any distinguishable absorption peak, implying a broad size distribution. This result indicates that the ligand concentration window for the formation of high-quality InP nanocrystals is narrow. Figure 1b shows the UV-Vis absorption spectra of NQDs prepared in a temperature range of 250°C to 280°C, using otherwise the parameters of the fixed molar ratio of In/MA to 3. It is evident that with increasing reaction temperature, the NQDs' excitonic peak is being red shifted, which implied the formation of bigger InP QDs. Figure 1c,d shows the UV-Vis absorption spectra of InP NQDs prepared using a new P precursor, P(SiMe_2_-*tert*-Bu)_3_, in a temperature range of 250°C to 280°C. It also shows that with increasing reaction temperature, the NQDs' excitonic peak is being red shifted.

**Figure 4:**
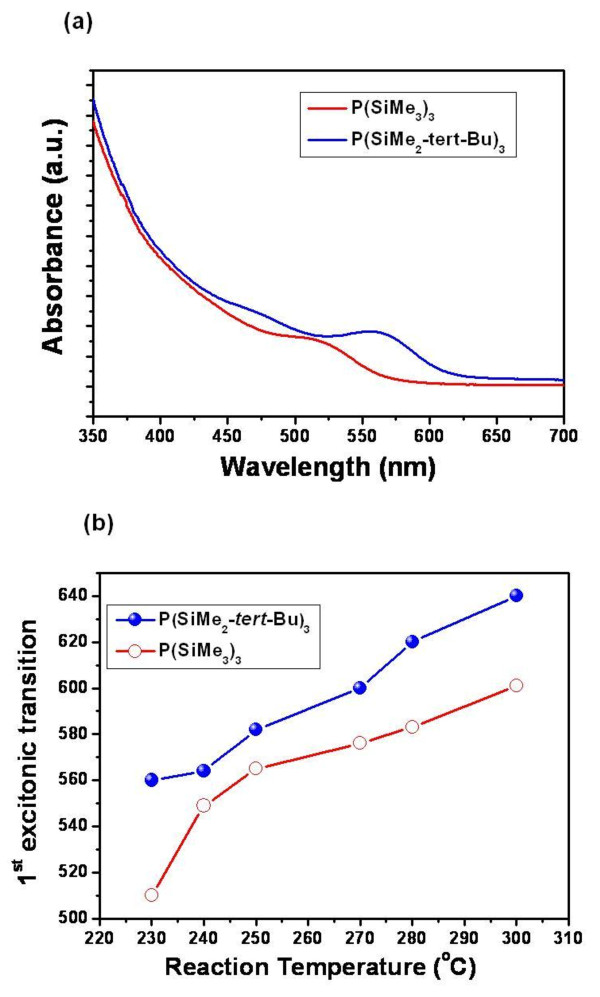
**UV-Vis absorption spectra of InP NQDs synthesized using various P precursors**. (**a**) UV-Vis absorption spectra of InP NQDs synthesized using different P precursors at the same temperature (injection at 230°C). (**b**) Changes in particle sizes indicated from the first excitonic transition in different reaction temperatures.

While phosphorus precursor **3 **did not undergo InP formation (see Table 1), precursor **2 **gave highly monodisperse crystalline InP NQDs when we used the same reaction protocol optimized for the P(SiMe_3_)_3 _precursor. By varying reaction temperature, we obtained an InP 1*s *absorption spanning from 560 nm to 640 nm. When compared with **1**, reactions using precursor **2 **resulted in a larger size as shown in Figure 4. At the same reaction temperature, InP synthesized using **2 **showed 20 to 40 nm more of red-shifted first excitonic transition (Figure 4b). Unlike II-VI NQDs, growth of InP nanocrystal is suggested as dominated by interparticle ripening. Increasing growth temperature does not expect to significantly broaden the excitonic feature. Rather, the myristic acid in the solution interferes with the ripening, thereby inducing broadening in excitonic transition [15]. We suggest that introducing the bulky *tert*-butyl group creates less nuclei at the same injection temperature and allows further growth using unreacted P precursors in the solution. With the previously used precursor **1**, we only obtained the dots with 1*s *absorption maxed at 580 nm in our reaction condition. The new P precursor with tertiary butyl group enabled a further shift to red up to 640 nm without compromising the size uniformity.

**Table 1:** Synthesis of InP NQDs using various P precursors.

P Precursors	InP (nm)	FWHM (nm)	Reaction temperature (°C)
P(SiMe_3_)_3 _(**1**)	495 to 601	49 to 60	230 to 300
P(SiMe_2_-*tert*-Bu)_3 _(**2**)	560 to 640	50 to 62	210 to 300
P(SiMe_2_Ph)_3 _(**3**)	No InP formation	-	-

FWHM, full width at half maximum.

**Figure 5:**
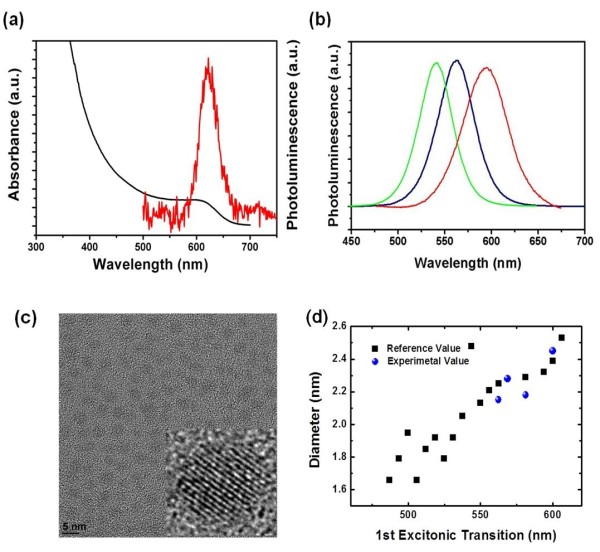
**Optical and structural characteristics of NQDs synthesized using new P precursors**. (**a**) Absorption and PL spectra of InP synthesized using P(SiMe_2_-*tert*-Bu)_3_. (**b**) Emission from InP/ZnS core-shell NQDs with a PL max of 535 nm (green line), 573 nm (blue line), and 625 nm (red line). (**c**) Electron micrograph of InP NQDs (*d *= 2.14 nm (*σ *= 0.38), 1*s *max = 560 nm). (**d**) Size of synthesized InP obtained from electron micrograph with respect to the first excitonic transition.

InP synthesized using new P precursors has poor band edge photoluminescence [PL] (Figure 5a) possibly due to surface traps, dangling bonds, and staking faults in the crystal as commonly seen in III-V NQDs compared with II-VI NQDs. Strategies to enhance the PL include chemically modifying the particle surface or epitaxial growth in a shell with a large bandgap material such as ZnS. *In situ *synthesis of InP/ZnS was reported with limited control in their sizes [7]. Here, we used a single-molecular ZnS precursor, diethyldithiocarbamate, for shell formation (Figure 3a). Slow addition of the air-stable ZnS precursor (diethyldithiocarbamate) at elevated temperature allowed shell formation on the surface of each particle, thereby enhancing PL properties (Figure 5b). The PL QY of the as-prepared InP NQDs was 1% (standard: Rhodamine 6G). The complete surface passivation procedure strongly enhanced the PL QY of the InP NQDs up to 18% to 28% (Figure 5b). Figure 5c shows a typical transmission electron microscope [TEM] image of an InP NC with mean diameters of 2.14 nm, exhibiting a quantum yield of approximately 28%. The diameters of at least 100 nanocrystals were counted for each sample from TEM images, and the average size and standard deviation were determined. Sizes of synthesized InP were able to be correlated with respect to the first excitonic transition, and they correspond well with a previously reported value synthesized with conventional P precursors [16].

In summary, we firstly developed a method for the synthesis of high-quality InP NQDs based on the use of synthesized P(SiMe_2_*-tert*-Bu)_3 _as the phosphorus precursor. A relatively stable phosphorus source allowed access to larger-sized InP NQDs without sacrificing a narrow size distribution. Further study of InP growth mechanism is under way.

## Conclusion

In this study, a novel and rapid method for the synthesis of high-quality InP NQDs was developed based on the use of *in situ *P(SiMe_2_-*tert*-Bu)_3 _as the phosphorus precursor. With respect to the conventionally used prescursor, the P(SiMe_3_)_3 _precursor is able to give access to larger-sized InP NQDs without sacrificing a narrow size distribution.

## Competing interests

The authors declare that they have no competing interests.

## Authors' contributions

The work presented here was carried out in collaboration among all authors. SMJ, SY, CSH, YK, and SJ defined the research theme. SMJ synthesized and characterized the indium phosphide NQDs and InP/ZnS. SY synthesized and characterized phosphorus precursors **1 **to **3**. SMJ and SY carried out the laboratory experiments and analyzed the data. YK, and SJ analyzed the data and discussed the analysis. YK, and SJ designed the experiments. YK and SJ wrote the manuscript. All authors read and approved the final manuscript.
